# Are Italian Newly Licensed Nurses Ready? A Study on Self-Perceived Clinical Autonomy in Critical Care Scenarios

**DOI:** 10.3390/healthcare12080809

**Published:** 2024-04-09

**Authors:** Giuseppe Stirparo, Pasquale Di Fronzo, Daniele Solla, Dario Bottignole, Luca Gambolò

**Affiliations:** SIMED (Società Italiana di Medicina e Divulgazione Scientifica), 43125 Parma, Italy; g.stirparo@areu.lombardia.it (G.S.); pasqualedifronzo89@libero.it (P.D.F.); daniele.solla@istitutotumori.mi.it (D.S.); dario.bottignole@unipr.it (D.B.)

**Keywords:** emergencies, clinical skills, nursing, health survey, life support care

## Abstract

The experience and self-confidence of healthcare professionals play critical roles in reducing anxiety levels during emergencies. It is important to recognize the potential impact of anxiety on performance. To enhance preparedness and confidence in managing emergencies, healthcare professionals benefit from regular training and simulations. Additionally, repeated exposure to emergency scenarios can help modulate physiological responses. Managing anxiety effectively is key, as heightened sympathetic stimulation associated with anxiety can adversely affect performance. This study aimed to investigate nurses’ self-assessed ability to manage emergency guidelines and their self-confidence in performing tasks in critical care settings. A questionnaire was provided to 1097 nurses. We compared the self-confidence of experienced nurses (ENs) and newly licensed nurses (NLNs) in managing emergency department shifts or critical patients, and found that ENs are more confident in these scenarios. This phenomenon was also observed in subjects who had taken simulation courses, although they were still a low percentage. Most NLNs feel sufficiently ready to work in medium-intensity wards. Attending advanced training courses enhances nurses’ self-confidence and may improve patient safety management., improving patient recovery, and minimizing errors. Attending courses improves the perception of autonomy of nurses in different scenarios.

## 1. Introduction

Critical care nurses should have advanced skills and the ability to manage critical scenarios. Performing optimal cardiopulmonary resuscitation (CPR) is one of the essential skills for nurses working in such a demanding setting. Timely and proficient CPR reduces the likelihood of death and complications following cardiac arrest [1]. Conversely, the absence of effective and prompt cardiopulmonary resuscitation results in lower rates of return of spontaneous circulation (ROSC) [2]. Consequently, promoting training and education in this field is imperative, as underlined by the European Resuscitation Council’s 2021 guidelines, which mandate that ‘high-quality resuscitation education is mandatory for healthcare providers at all levels’ and emphasize the necessity of ongoing education to ensure adequate preparedness in critical situations [3].

Sometimes, nurses are the first healthcare staff patients meet in acute care settings. The interpretation of the ECG plays a vital role in initiating and facilitating appropriate care escalation and interventions [4,5].

Advanced Life Support (ALS) is the gold standard for assessing competence in advanced resuscitation. Younger candidates, participants in emergency disciplines, and good pre-course resuscitation knowledge are the most important predictors of ALS provider courses’ success [6].

In Italy, the basic nursing education curriculum often lacks specific courses related to critical care, and only Basic Life Support and Defibrillation (BLSD) courses are included. However, nursing education incorporates a specific class on first aid [7]. After graduating from the bachelor’s degree program, nurses can work in any hospital setting. However, the opportunity to attend internships in highly specific and intensive care units, such as the emergency room, resuscitation/intensive care, and semi-intensive care, is low. Therefore, nurses who are newly hired or new to the work environment may find it difficult or feel unprepared to deal with such a specific work path. Cardiac arrest is a medical emergency whose chances of survival can be increased by rapid cardiopulmonary resuscitation (CPR) and early use of an automated external defibrillator (AED).

Basic Life Support (BLS) training became mandatory in Italy to spread knowledge of resuscitation maneuvers in the workplace [8] but is still optional for nurses and medical doctors. ACLS (Advance Cardiovascular Life Support) courses are now one of the standard learning practices most spread around the world because of the great satisfaction of nurses and doctors [9] and the impact of health professional knowledge [6]. 

Training is important in critical care, and nurse educators in acute care settings should consider training and education to maintain nurses’ competency during their time there. There is an important educational and clinical need to more clearly define the skills of nurses in ECG [10]. Continuing professional education (CPE) is essential to develop, maintain, and update professional skills and practice, providing a high standard of patient care [11,12]. CPE offers the opportunity to healthcare workers to stay engaged in evidence-based practice and best practice guidelines and to update their clinical skills [13].

This research aims to investigate nurses’ self-assessed ability to adhere to international medical emergency guidelines and their self-confidence in performing routine tasks within specialized critical care settings. The study places particular emphasis on comparing these factors between newly licensed nurses (NLNs), defined as nurses who graduated less than 12 months prior to the study, and experienced nurses (ENs), encompassing all other nurses. Additionally, the research aims to identify influential factors that may contribute to variations in self-assessment and confidence levels among nurses.

## 2. Materials and Methods

### 2.1. Study Design and Setting

This study followed a cross-sectional design. The target population consisted of licensed nurses attending training courses.

### 2.2. Sample and Data Collection

Paper-based questionnaires were distributed during multiple training sessions focused on critical care and emergency medicine. Instructors handed out the questionnaires to all 1097 participating nurses during the SIMED training courses conducted between June 2021 and December 2022. Nurses were requested to complete these questionnaires after the introductory segment but before the commencement of the training sessions. Anonymity was preserved by abstaining from soliciting sensitive information (such as names, surnames, and dates of birth), and privacy was guaranteed to participants when completing the questionnaire. The population was represented by convenience sampling. The participants expressed their consent before administration of the questionnaire; those who did not want to participate in the survey were excluded from the study without consequence to their course. After excluding missing and partially completed questionnaires, the data were reviewed. 

### 2.3. Instruments

There are no universally agreed quantitative criteria in the literature for a cut-off point of experience to define a nurse expert, so we decided to utilize a cut-off point used by Bellini et al. for other healthcare workers (physicians) [14]. We defined the population of nurses who achieved graduation in the previous twelve months as “newly licensed nurses” (NLNs), also referring to stage 2 of Benner’s theory [15].

SIMED group produced the questionnaire ad hoc for this study, and it was inspired by a study in which the sample consisted of physicians [14] and adapted to the nursing environment. The questionnaire was divided into 3 sections. The first included a demographic module. The second consisted of 5 closed questions with a dichotomous response of “yes/no” for the perceived ability to interpret an ECG, to manage a heart attack according to the BLSD guidelines, to manage a cardiac arrhythmia according to the ACLS guidelines, to manage trauma in the pre-hospital setting according to ITLS guidelines, and to perform airway management. The third section included 5 self-assessment questions about different scenarios: an emergency ward (EM), a medium-intensity ward (MI), and working in an Accident and Emergency Unit (A&E). To allow better data stratification, the answers in this last section were classified according to a 5-point Likert-type scale, where 1 stood for “poor confidence” and 5 stood for “full confidence” [16]. For the analysis, we decided to dichotomize the answers into 2 groups: “0”, when nurses chose numbers 0–3 on the Likert scale (i.e., they did not feel sufficiently ready), and “1”, when they chose numbers 4–5 on the Likert scale (i.e., they felt sufficiently ready). People who felt confident in all scenarios were categorized as fully confident (FULL).

### 2.4. Data Analysis

Continuous variables were summarized using the mean and standard deviation (SD), whereas categorical values are presented as numbers and percentages. We set the alpha level at 0.05, so all *p*-values below the threshold level were considered statistically significant.

Software R version 4.1 for Microsoft Windows was chosen to perform all statistical analyses. Continuous variables were compared between NLNs and ENs utilizing *t*-tests, whereas categorical variables were assessed using chi-square tests. Additionally, a logistic regression analysis was performed to ascertain the predictive capacity of the variables on achieving self-perceived full autonomy.

## 3. Results

The survey was administered to 1097 nurses: 918 questionnaires were completed. A total of 912 questionnaires were deemed suitable for analysis, with a response rate of 83.7%. In this sample, 685 (75.1%) were collected from ENs, and 227 (24.9%) from NLNs. The total sample can be viewed in Table 1. There are no statistical differences in the sex distribution of the participants (*p* = 0.581).

In this sample, most of the NLN respondents (85.5%) felt confident in managing a cardiac arrest by applying BLSD guidelines followed by ECG interpretation. Inability reached elevated percentages (all greater than 60%) for four out of five questions about self-perception, except for managing BLSD procedures (questions 1, 3, 4, 5). The worst self-perceived clinical autonomy was reported in trauma management (23.3%). On the other side, ENs felt confident with BLSD knowledge, and this was the same in the NLN population. Still, they declared that they had more difficulty in managing pre-hospital trauma according to ITLS guidelines and dealing with cardiac arrhythmias according to ACLS guidelines (77.5% and 63.4%, respectively). All results are shown in Table 2.

We used the chi-square test and OR to verify the hypothesis that self-reported ECG, BLSD, and ACLS knowledge and ability to use guidelines were substantially different in the two groups. We obtained a statistically significant association (ECG: *p* = 0.0024, OR, 1.43 [95% CI, 1.05–1.95]; BLSD: *p* = 0.006, OR, 1.87 [95% CI, 1.19–2.96]; ACLS: *p* = 0.01, OR, 1.54 [95% CI, 1.11–2.14]). 

A low number of NLNs felt sufficiently ready to work in the different scenarios: the majority declared to feel sufficiently ready to work in medium-intensity wards (22.02%), and a reasonably low number declared to be able to manage all scenarios (3.96%). In the EN group, 21.9% declared to feel ready to work in MI wards and only 12.99% in all scenarios. All the results of singular scenarios are shown in Table 3. All data were statistically significant with a *p*-value < 0.05.

Logistic regression analysis assessed the predictive factors that influence the attainment of full autonomy in response to the presented scenarios. The four main predictors considered were gender, age, experience, and courses attended. Table 4 presents the logistic regression results for predicting self-perceived full autonomy, examining the potential impact of gender, age, experience level, and course attendance on awareness across various scenarios in the study.

## 4. Discussion

In our study, we defined the target population as nurses who have graduated within the past 12 months, referring to them as “newly licensed nurses” (NLNs). To the best of our knowledge, our questionnaire is the first of its kind to assess self-perceived clinical autonomy in specific scenarios among NLNs. For the development of the questionnaire, we referred to a study intended for physicians [14], with adaptations tailored to the nursing population.

As anticipated, a greater proportion of experienced nurses attended the courses and were familiar with all presented guidelines, except pre-hospital trauma. Moreover, a notably higher percentage of experienced nurses reported feeling adequately prepared for various scenarios and expressed a sense of full autonomy in their practice.

Regarding heart attack management, our NLN sample showed relatively high confidence in the training received, with 85.5% of respondents stating their familiarity with BLSD. In contrast, more than 70% of respondents could not manage advanced emergencies such as airway emergencies, pre-hospital trauma, and arrhythmia. The reason for this might be the absence of specific courses in ACLS and ITLS in the academic nursing curriculum. Conversely, the strong knowledge of BLSD could be due to the wide dissemination of courses for nurses and lay people too [17]. The inclusion of BLSD principles in the nursing curriculum, particularly in the first aid class, may also contribute to this proficiency [7].

Over 50% of the whole sample did not know how to interpret an ECG. This aligns with the literature, which highlights that nurses identified a lack of regular training in and insufficient exposure to the interpretation of electrocardiograms. Therefore, standard training and education are recommended [10]. This suggests the importance of training in the interpretation of electrocardiograms [18], including for nurses working in out-of-hospital emergencies and A&E, who must have good electrocardiogram knowledge. 

In total, 73% of NLNs did not know ACLS guidelines; this could be acceptable in this small population, but, surprisingly, more than 63% of ENs did not know them either. 

Our survey revealed a substantial knowledge gap among NLNs regarding the management of pre-hospital trauma. Unfortunately, we do not have data about the knowledge of this item in the population of out-of-hospital care nurses.

A systematic review of ATLS training support was performed by Abu Zidan et al., showing its usefulness in enhancing knowledge [19]. 

An important contribution of the questionnaire to the study is the self-perception of NLNs managing some specific scenarios. Most of them declared a low level of confidence across all scenarios. Only 20% of NLNs felt confident in working in a medium-intensity ward. These results suggest the importance of training and continuous education, particularly with ACLS and ITLS and airway management. We can see that both ENs and NLNs were confident applying BLSD, suggesting that mandatory BLSD, ACLS, and ITLS courses could help standardize behaviors and increase self-perception. This study also indicates that ENs, for example, felt more confident to work in A&E than did NLNs, with an OR of 1.66 [95% CI, 1.12–2.45]. Noteworthy is the astonishing result that only a small percentage of ENs (12.99%) felt confident in working in the FULL scenarios. It is important to conduct further studies (for example, qualitative studies) to investigate why expert nurses do not feel ready for all scenarios despite long experience. The result of the study could be in line with the result of an Italian study where the fatigue condition in nurses and the variables such as job role, night shift, and years of work experience were considered [20]. Nowadays, nurses and physicians are leaving emergency rooms, suggesting that workload affects choices after many years.

At the same time, 92.07% of NLNs declared that they do not feel confident in managing patients in an EM and 83.7% of NLNs in A&E; this low self-perception could be due to the lack of internships in these areas or to the absence of monothematic courses attended. For this reason, we remark on the importance of the aforementioned courses at university. 

The logistic regression models presented in this study not only provide valuable insights into the factors influencing full autonomy among nurses but also quantify the extent to which these factors contribute to predicting the likelihood of achieving a perception of full autonomy (12.97%). 

Examining the individual predictor coefficients in the models, it becomes clear that training courses, such as ECG, ACLS, and ITLS, play a fundamental role in predicting full autonomy. BLSD probably gives a sense of autonomy to nurses because it is a widely attended course and it is also taught at the university. 

Nurses who completed the aforesaid courses exhibit significantly higher odds of perceiving full autonomy in their practice. These courses contribute substantially to the overall predictive power of the models. Completing these courses not only provides essential clinical skills but also raises confidence and competence in managing complex scenarios. These data further support the importance of attending training courses to increase professional autonomy and maintain skills over time. 

Self-confidence among healthcare workers may positively influence their performance [21,22,23]. Additionally, a confident attitude can reduce stress and anxiety, leading to better mental health and job satisfaction among workers, probably regulating the autonomic system [24,25]. The theme of self-confidence remains important to ensure better performance for patients and to provide safety to operators. It is crucial to reduce the state of anxiety and agitation in operators who may find themselves managing emergencies. The regulation of anxiety may be due to neuromodulation, which can be reduced, according to the analysis presented by our study, through two methods: simulation and professional experience. This is because stimulating neuromodulation can lead to a situation of stimulus adaptation, resulting in a reduction in its sensitivity [25,26].

In a study performed on anesthetist nurses, after brief ACLS training, knowledge and skills were significantly improved, but knowledge was not retained at the post-test levels until the three-month check, whereas skills had persisted. Patient survival to discharge after resuscitation was measured in a cohort case–control study. As reported in the literature, the ROSC rate is higher if nurses are trained in ACLS [27]. The study by Smith et al. showed a decline in skill retention with nurses unable to perform ACLS and BLS skills at standard levels for the entire certification period. More frequent refresher training was needed [28]. 

The evidence supports the need for ACLS training for critical care nurses, organized ongoing refresher courses, multidisciplinary practice using technologically advanced simulator mannequins, and videotaped reviews to prevent knowledge and skill degradation for effective resuscitation efforts [29,30].

Our results should be viewed considering some limitations. First, the presence of selection bias due to the reliance on nurses who participated in training courses potentially skewed the sample towards individuals more interested in critical care scenarios and restricts the generalizability of the findings to broader nurse populations. There is also a risk of response bias, whereby participants may provide socially desirable responses rather than honest reflections of their experiences. Additionally, the assessment of readiness in critical care scenarios does not encompass all challenges encountered in real-world clinical practice. The reliance on subjective self-assessment may not always align with nurses’ actual clinical competencies. The lack of explicit validation regarding the questionnaire’s psychometric properties raises questions about its effectiveness in accurately measuring readiness and autonomy. The sample exhibited a mild gender disproportion, with a higher percentage of female professionals compared to male professionals. This gender disparity could introduce biases in the study outcomes, as gender-related factors might influence nurses’ readiness and confidence levels in critical care situations differently. Lastly, the research was conducted within a specific geographical region, which might not fully represent the diversity of nurses across different healthcare settings and regions.

To address these limitations and areas for improvement, future research could consider implementing multivariate analysis to explore the combined effects of various variables on nurses’ readiness and autonomy levels. Additionally, external validation through replication studies in diverse healthcare settings or regions would enhance the generalizability of the findings. Moreover, efforts to validate the questionnaire’s psychometric properties and minimize response bias would strengthen the validity and reliability of the study’s findings.

## 5. Conclusions

In conclusion, this study highlights significant differences between newly licensed nurses and experienced nurses in their self-assessed confidence and preparedness in various critical care scenarios. While NLNs demonstrated confidence in Basic Life Support, they exhibited lower confidence levels in managing advanced emergencies like airway emergencies, pre-hospital trauma, and arrhythmias. A larger number of experienced nurses participated in the courses and were familiar with all the presented guidelines, except for pre-hospital trauma. As expected, a significantly higher percentage of experienced nurses reported feeling well prepared for different scenarios and expressed a sense of complete autonomy in their practice.

Notably, the completion of training courses such as ECG, ACLS, and ITLS emerged as a crucial predictor of nurses’ perception of full autonomy in practice, highlighting the importance of continuous education in enhancing clinical skills and confidence levels. 

Self-confidence among healthcare workers not only influences performance but also contributes to improved mental health and job satisfaction. Addressing these findings necessitates a comprehensive approach involving curriculum enhancements, mandatory training courses, and ongoing professional development initiatives to ensure nurses are adequately equipped to handle complex scenarios and deliver optimal patient care with confidence and competence.

## Figures and Tables

**Table 1:** The demographic and working data of participants, both in total and grouped by experience.

	Total	ENs	NLNs
**Number (%)**	912	685 (75.1%)	227 (24.9%)
**Age mean (SD)**		34.5 (9.13)	26.8 (6.12)
**Age minimum (years)**		21	20
**Age maximum (years)**		62	53
**Sex (F)**	715 (78.4%)	540 (75.5%)	175 (24.5%)
**Months of job mean (SD)**		105 (99.5)	5.96 (4.59)

**Table 2:** Survey results in emergency management; results are for both ENs and NLNs.

	Experience	
	ENs	NLNs
Questions	Yes (n; %)	Yes (n; %)
Do you think you can interpret an ECG?	(306; 44.7%)	(82; 36.1%)
2. Are you able to manage a cardiac arrest according to BLSD guidelines?	(628; 91.7%)	(194; 85.5%)
3. Are you able to manage arrhythmia according to ACLS guidelines?	(251; 36.6%)	(62; 27.3%)
4. Are you able to manage pre-hospital trauma according to ITLS or ATLS guidelines?	(154; 22.5%)	(53; 23.3%)
5. Are you able to perform advanced airway management?	(318; 46.4%)	(68; 30%)

**Table 3:** Survey results in scenario management; results are expressed for both ENs and NLNs. *p*-values, Odds Ratio (OR), and Confidence Interval at 95% (95% CI) are reported too.

	Experience					
	ENs	NLNs	*p*	OR	95% CI	
**self-perceived autonomy EM**						
yes	158	18	<0.001	3.48	2.08	5.82
no	527	209				
**self-perceived autonomy MI**						
yes	272	50	<0.001	2.33	1.64	3.31
no	413	177				
**self-perceived autonomy A&E**						
yes	167	37	0.011	1.66	1.12	2.45
no	518	190				
**FULL**						
yes	89	9	<0.001	3.62	1.79	7.30
no	596	218				

**Table 4:** Logistic Regression Analysis of Self-Perceived Full Autonomy in Responding to Presented Scenarios.

Predictor	Estimate	SE	Z	*p*	OR
Intercept	−5.13541	0.6720	−7.9642	<0.001	0.006
Sex					
M-F	0.09610	0.2716	0.354	0.723	1.101
Age	0.00858	0.0134	0.640	0.522	1.009
Experience					
EN-NLN	0.92846	0.3123	2.973	0.003	2.531
ECG					
Y-N	1.14696	0.2741	4.185	<0.001	3.149
ITLS					
Y-N	1.19864	0.2497	4.801	<0.001	3.316
BLSD					
Y-N	0.51916	0.5067	1.025	0.306	1.681
ACLS					
Y-N	0.90635	0.2651	3.419	<0.001	2.475

## Data Availability

The data that support the findings of this study are available on request from the corresponding author.
